# Semicontinuous sophorolipid fermentation using a novel bioreactor with dual ventilation pipes and dual sieve‐plates coupled with a novel separation system

**DOI:** 10.1111/1751-7915.13028

**Published:** 2017-12-13

**Authors:** Yaguang Zhang, Dan Jia, Wanqi Sun, Xue Yang, Chuanbo Zhang, Fanglong Zhao, Wenyu Lu

**Affiliations:** ^1^ School of Chemical Engineering and Technology Tianjin University Tianjin 300350 China; ^2^ Key Laboratory of System Bioengineering Ministry of Education Tianjin University Tianjin 300350 China; ^3^ SynBio Research Platform Collaborative Innovation Center of Chemical Science and Engineering (Tianjin) Tianjin University Tianjin 300350 China; ^4^ Department of Chemical and Biological Engineering The University of Alabama 285 Hardaway Hall, 401 7th Avenue Tuscaloosa AL 35487 USA

## Abstract

Sophorolipids (SLs) are biosurfactants with widespread applications. The yield and purity of SLs are two important factors to be considered during their commercial large‐scale production. Notably, SL accumulation causes an increase in viscosity, decrease in dissolved oxygen and product inhibition in the fermentation medium. This inhibits the further production and purification of SLs. This describes the development of a novel integrated system for SL production using *Candida albicans* O‐13‐1. Semicontinuous fermentation was performed using a novel bioreactor with dual ventilation pipes and dual sieve‐plates (DVDSB). SLs were separated and recovered using a newly designed two‐stage separation system. After SL recovery, the fermentation broth containing residual glucose and oleic acid was recycled back into the bioreactor. This novel approach considerably alleviated the problem of product inhibition and accelerated the rate of substrate utilization. Production of SLs achieved was 477 g l^−1^, while their productivity was 1.59 g l^−1^ h^−1^. Purity of SLs improved by 23.3%, from 60% to 74%, using DVDSB with the separation system. The conversion rate of carbon source increased from 0.5 g g^−1^ (in the batch fermentation) to 0.6 g g^−1^. These results indicated that the integrated system could improve the efficiency of production and purity of SLs.

## Introduction

Sophorolipids (SLs) are amphiphilic compounds produced from a variety of saccharides and vegetable oils using the yeast *Starmerella bombicola* and its related strains (Rosa and Lachance, 1998). Widely known as extracellular biosurfactants (Rashad, 2014), SLs are used as antimicrobial agents (Borsanyiova *et al*., 2016), biopharmaceuticals (Singh *et al*., 2016), nanomaterials (Pandey *et al*., 2016), emulsifying agents (Liu *et al*., 2009), cosmetic ingredients (Gharaei‐Fathabad, 2011) and detergents (Lee *et al*., 2014). The economic value and market demand for SLs has increased on account of their high detergency, low cytotoxicity and biodegradability (Lee *et al*., 2008).

The use of SLs has also gained much attention due to increasing environmental concerns. However, detailed studies are required for improving the large‐scale production of SLs (Ribeiro *et al*., 2013). Recent studies on SLs have focused on utilizing cheap substrates (Shah *et al*., 2007; Solaiman *et al*., 2014) and improving production efficiency (Daverey and Pakshirajan, 2009). Several substrates were explored for replacing glucose in the SL fermentation medium, such as glycerol (Bhangale *et al*., 2014), sugarcane molasses (Takahashi *et al*., 2011) and corncob hydrolysate (Konishi *et al*., 2015). Maddikeri *et al*. (2015) produced 55.6 g l^−1^ SLs from waste frying oil using ultrasound technology. Bajaj and Annapure (2015) produced a novel SL (40.24 g l^−1^); however, its yield was low because microbial growth was inhibited by the second carbon source used during fermentation. In another study, SL production reached 280 g l^−1^ using deproteinized whey and canola oil as substrates (Daniel and Syldatk, 1998). High SL production (422 g l^−1^) was achieved using a two‐stage fed‐batch process (Kim *et al*., 2009). It was reported that crude SL production reached 365 g l^−1^ in 8 days using the feeding‐rate‐controlled fed‐batch cultivation technique (Rodrigues *et al*., 2006).

The UDP‐glucosyltransferases UgtA1 and UgtB1 are responsible for the biosynthesis of SLs from hydroxylated fatty acids. It has been reported that both glucosyltransferases were inhibited by their product SLs. Further, when the product concentration reached > 1 mM, the residual activity observed for UgtA1 was only 62% (Saerens *et al*., 2011a,b). Moreover, Haque *et al*. (2016) observed that SLs had an inhibitory effect on biofilm formation and hyphal growth in *Candida albicans*. It was observed that SLs downregulated the expression of hypha‐specific genes such as *HWP1*,* ALS1*,* ALS3*,* ECE1* and *SAP4*. About 80% cell growth was inhibited by 60 μg ml^−1^ SLs (minimum inhibitory concentration). For the large‐scale production of highly pure SLs, product inhibition during SL fermentation needed to be addressed. In this regard, several studies have shown that coupling the fermentation and separation processes using innovative bioreactor technologies was an effective strategy (Nguyen *et al*., 2011).

Our study showed that SLs had an inhibitory effect on the growth of *C. albicans* O‐13‐1. In fact, they could be consumed by *C. albicans* O‐13‐1 as a carbon source. This study aimed to establish an integrated fermentation and separation process for achieving high‐level high‐purity SL production by alleviating the problem of product inhibition and recycling the fermentation broth. A novel bioreactor with dual ventilation pipes and dual sieve‐plates (DVDSB) was developed for improving SL production. In addition, our newly designed two‐stage separation system circumvented the problem of product inhibition, and enabled recycling of the residual substrate and cells. By using this novel technique, we achieved high efficiency in the fermentation process, coupled with the simultaneous separation of SLs.

## Results and Discussion

### Batch and fed‐batch SL fermentation

**Figure 1:**
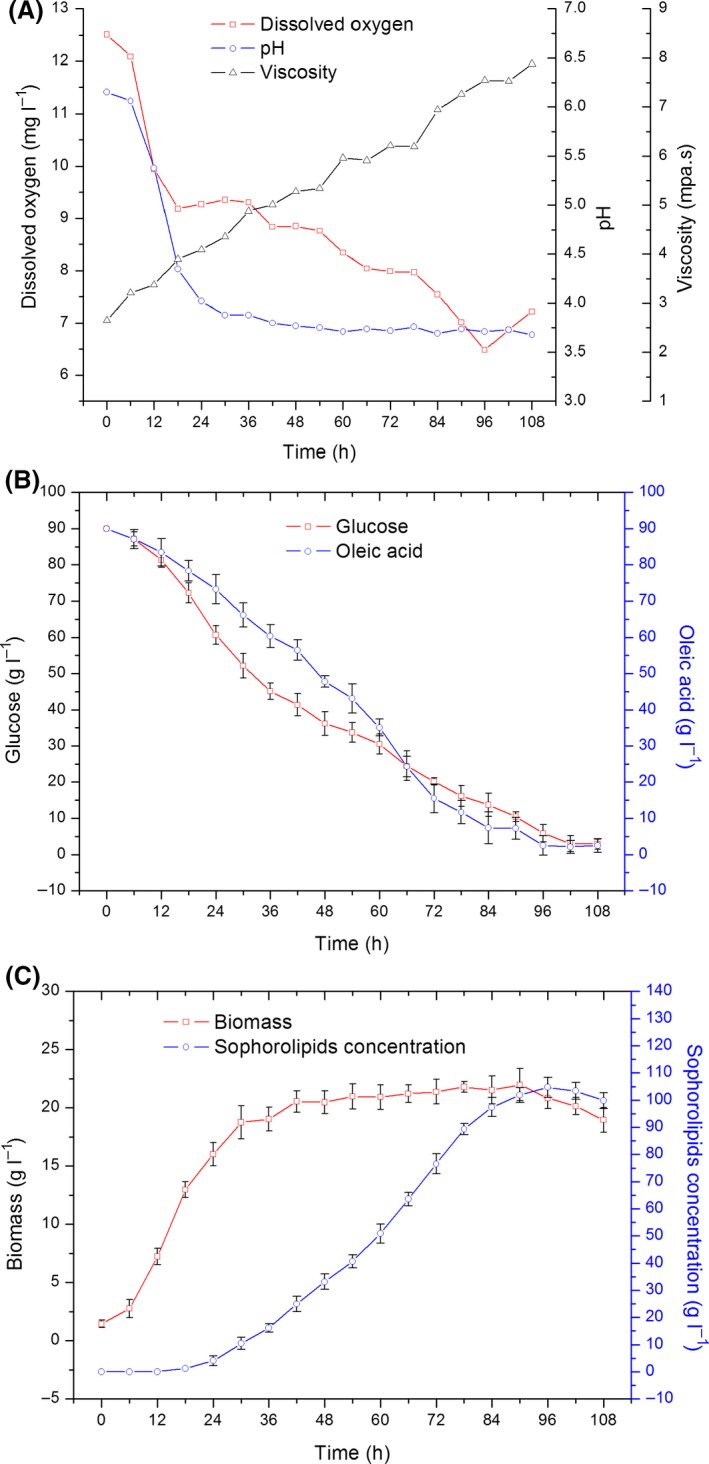
Fermentation curves during batch fermentation over a 96‐h period. A. Time‐course of dissolved oxygen (DO), viscosity and pH. B. Consumption of glucose and oleic acid substrates. C. Cell growth and SL production. The results shown are the mean values from triplicate experiments.

Orthogonal experiments (Tables S1 and S2) for optimizing the fermentation conditions (such as pH, temperature, quantity of inoculum and stirring speed) for a 5‐l batch fermentation were conducted, which aimed at achieving maximum cell concentration and SL production. After 96 h of batch fermentation (Fig. 1), the production of SLs reached 104.7 g l^−1^ with a productivity of 1.09 g l^−1^ h^−1^. The maximum cell concentration was 21.98 g l^−1^, which was determined using orthogonal experiments. As fermentation proceeded, the viscosity of broth considerably increased from 2.34 to 8.35 mpa s^−1^. Additionally, the rate of substrate utilization reduced significantly with the accumulation of SLs. SL production was considerably affected by varying the amounts of both carbon sources. (Fig. S1) It has been observed that the optimum substrate concentration for SL fermentation was 30–90 g l^−1^ glucose and 30–60 g l^−1^ oleic acid (Kim *et al*., 2009). Notably, the optional range of substrates must be strictly controlled.

**Figure 2:**
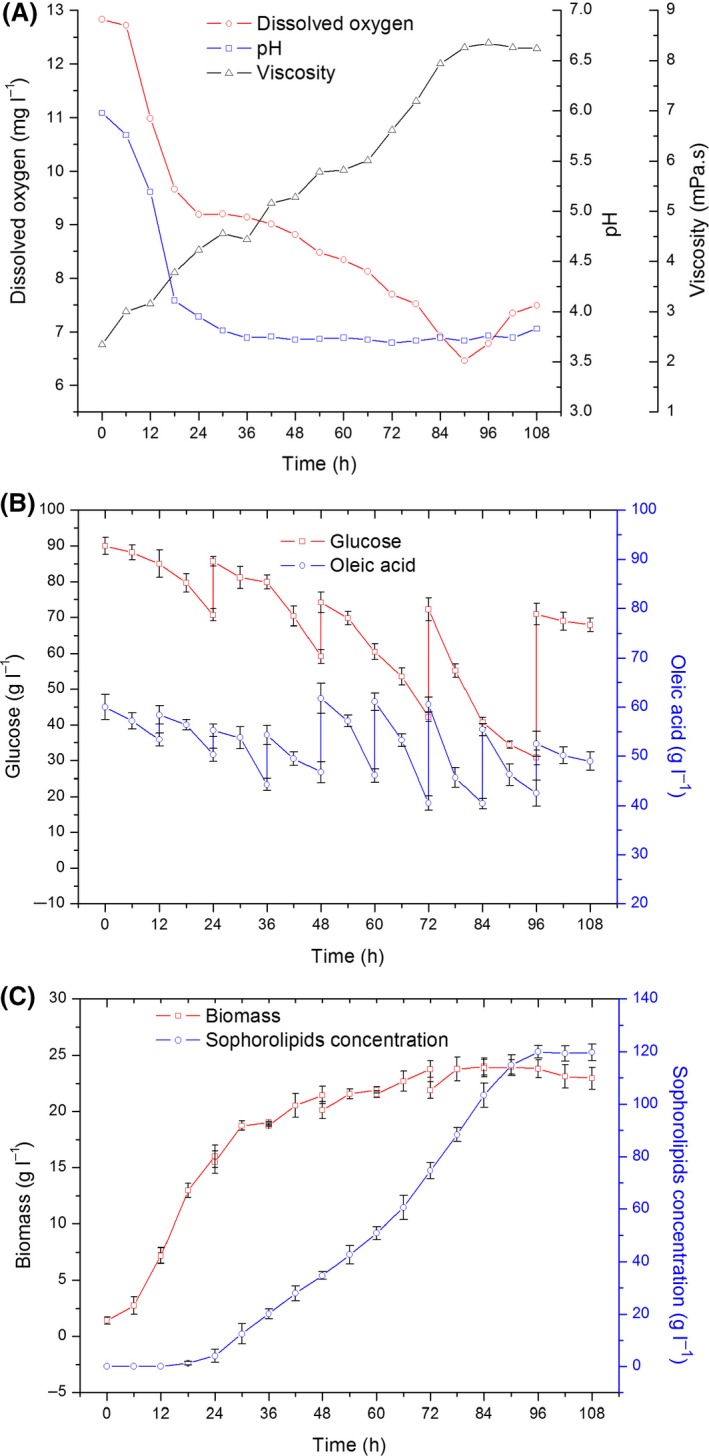
Fermentation curves during fed‐batch fermentation over a 96‐h period. A. Time‐course of DO, viscosity and pH. B. Consumption of glucose and oleic acid substrates. C. Cell growth and SL production. The results shown are the mean values from triplicate experiments.

The fed‐batch SL fermentation in a 5‐l bioreactor was conducted to alleviate the aforementioned problems (Fig. 2). The carbon source was added, and its concentration was monitored in real time, as described in the Experimental Procedures section. The production of SLs improved by 14.3% and reached 120 g ^−1^ after 96 h. Maximum cell concentration increased by 9.2% compared with the batch fermentation and reached 24.02 g l^−1^. Although the optimum substrate concentration was used, after 96 h, the cell concentration decreased as the broth viscosity increased. These results indicated that the accumulation of SLs may cause the product inhibition effect, which restricts the cells from converting the substrates to SLs. Thus, we proceeded to evaluate the inhibitory effects of SLs on the growth of *C. albicans* O‐13‐1.

### Inhibitory effects of SLs on the growth of *C. albicans* O‐13‐1

Few studies have reported the inhibitory effects of SLs on the growth of microbial cells. To determine the minimum inhibitory concentration, different concentrations of SLs (0–95 g l^−1^) were added to slant medium in petri dishes. This tolerance test indicated slow cell growth on the medium containing 35 g l^−1^ SLs (Fig. S2). Moreover, the yeast liquid culture diluted 10^3^ and 10^4^ times showed considerably fewer colonies as the SL concentration increased (especially at SL concentrations > 75 g l^−1^).

**Figure 3:**
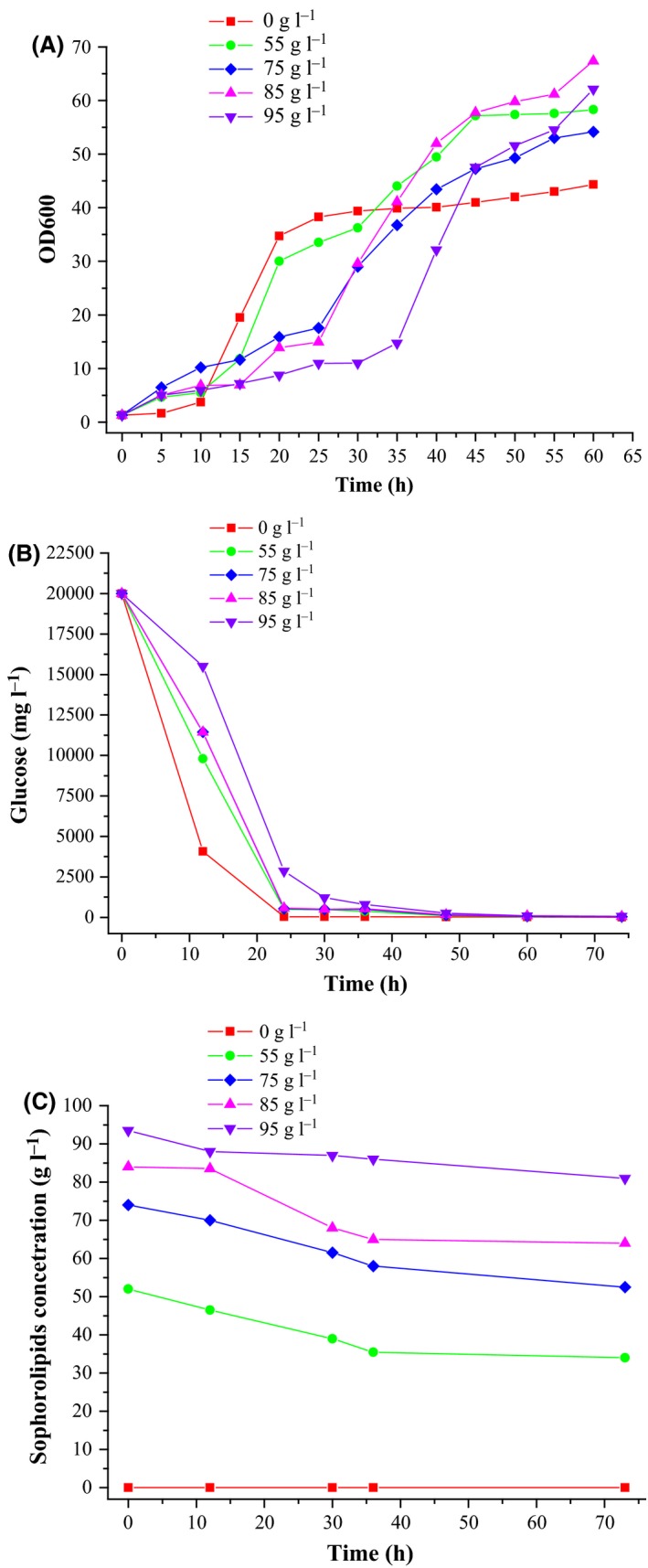
Inhibitory effect of added SLs on growth of *C. albicans* O‐13‐1. A. Time‐course of OD_600_ *C. albicans* O‐13‐1 without SL‐producing ability. B. Consumption of glucose. C. SL concentration.

Based on these results, flask fermentation for validating SL‐mediated inhibition was conducted (Fig. 3). During the initial period of fermentation (< 30 h), following a gradual increase in SL concentration, the cell concentration markedly decreased and a delay in cell growth was observed. Furthermore, glucose consumption in the fermentation broth also decreased, which was consistent with the pattern of growth curve of cells. After 30 h, the cell growth in experimental groups exceeded that in the control group and was subsequently stabilized, which indicated that SLs were used for cell growth during fermentation. As shown in Fig. 3c, a decrease in the residual SLs suggested that SLs could be utilized as a carbon source during their own production. Thus, SLs suppressed cell growth during the fermentation prophase; however, they could be utilized again as the carbon source for cell growth. These results indicated that product separation was mandatory to alleviate the problem of product inhibition. Thus, a concurrent method for SL recovery during fermentation was developed.

### A novel bioreactor (DVDSB) for semicontinuous fermentation

**Figure 4:**
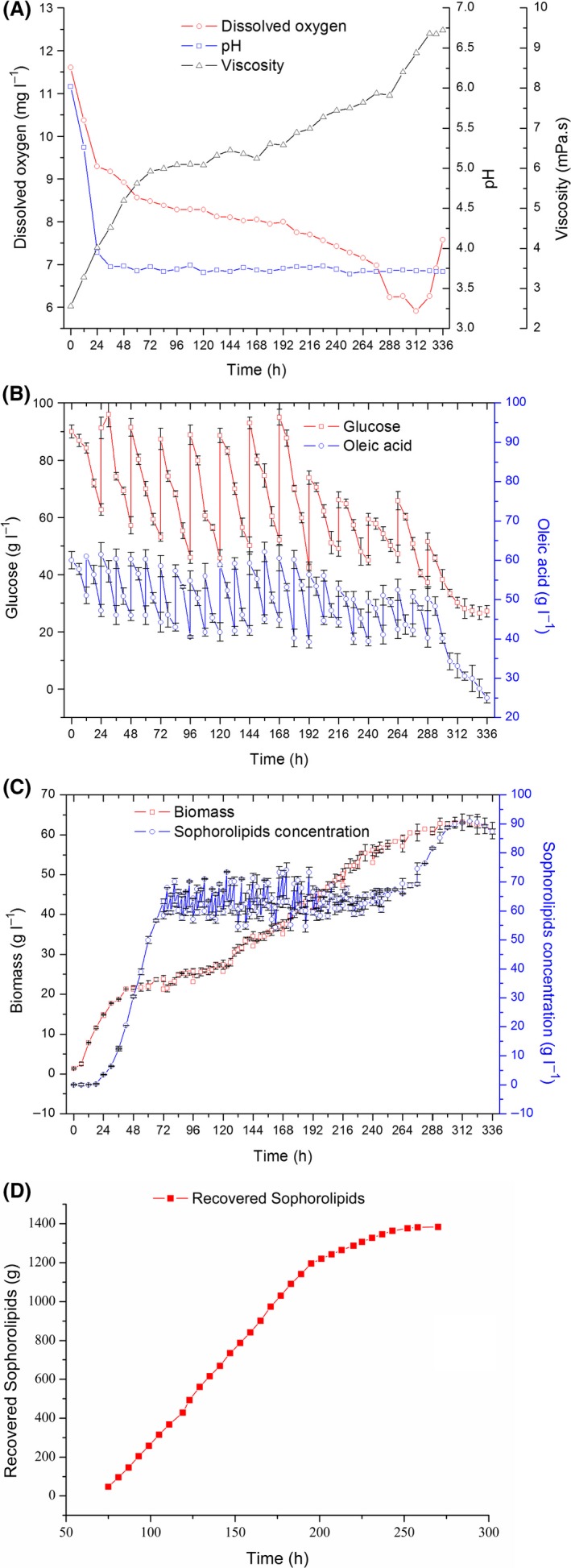
Fermentation curve during semicontinuous fermentation over a 320‐h period. A. Time‐course of DO, viscosity and pH. B. Consumption of glucose and oleic acid substrates. C. Cell growth and SL production. D. Total quantity of recovered SL. The results shown are the mean values from triplicate experiments.

Results showing substrate consumption, product accumulation and other fermentation parameters during semicontinuous SL fermentation using the novel DVDSB system have been summarized in Fig. 4. The schematic (Fig. S5) and detailed design description of DVDSB have been shown in the Supporting information.

During the initial stages of fermentation, both ventilation pipes were open, and both chambers of the bioreactor were involved in the fermentation process. During the first 70 h, the performance of semicontinuous fermentation was similar to that of the conventional fed‐batch fermentation. At 70 h, the SL concentration reached 60.65 g l^−1^, and product separation was performed at 3–6 h intervals by closing the main oxygen supply pipeline. As the upper ventilation pipe remained open, fermentation proceeded independently in the cylindrical area. Notably, the time for SL sedimentation considerably decreased to 10–15 min. Additionally, the sedimentation distance decreased from a height of 22.4–8 cm in the cone‐shaped camber.

Sophorolipids concentration in the DVDSB was maintained at 55–75 g l^−1^ (Fig. 4c), indicating that removal of the product maintained optimum fermentation conditions. Compared with the conventional fermentation process, viscosity of the fermentation broth was lower in DVDSB, while the dissolved oxygen (DO) content was higher (Figs 1a and 4a). The fermentation process concluded at 320 h, during which time, the maximum cell concentration increased to 61.76 g l^−1^, which suggested that the metabolic activity of cells was prolonged due to the removal of SL and feeding with supplementary medium. The problem of product inhibition was alleviated by using DVDSB. The titre of SLs produced by semicontinuous fermentation with DVDSB was 484 g l^−1^ with a productivity of 1.51 g l^−1^ h^−1^. Compared with the traditional fed‐batch fermentation, DVDSB increased SL productivity by 20.8%. The total amount of SL recovered was 1383 g, as shown in Fig. 4d; however, it was also observed that the SLs could not be excluded out of the fermenter after 276 h. The substrate conversion efficiency was 0.6 g g^−1^, compared with 0.5 g g^−1^ for the original fed‐batch process.

However, certain problems could not be resolved. The purity of SLs was only 60%. Meanwhile, 40% fermentation broth was removed from DVDSB along with the discharge of SLs, resulting in low availability of the microbial culture (yeast). To improve the purity of SLs and reduce the wastage of fermentation broth (or ensure yeast recycling), we introduced a separation system.

### Design of the two‐stage separation system with DVDSB

Edible oil is generally used as the hydrophobic carbon source for SL production. During fermentation, we observed that genetically modified soybean oil (GMSO) was miscible with SLs, formed a hydrophobic phase, and separated from the fermentation broth phase within 1 min (Fig. S3). Thus, we hypothesized that GMSO could be used as a phase separating agent for the discharged SLs. To simulate the fermentation environment in real time, different ratios of SLs and GMSO (5:1, 2:1, 1:1, 1:2 and 1:5) were mixed with equivoluminal broth. Interestingly, the mixture having 2:1 ratio layered particularly clearly after settling for 15 min.

**Figure 5:**
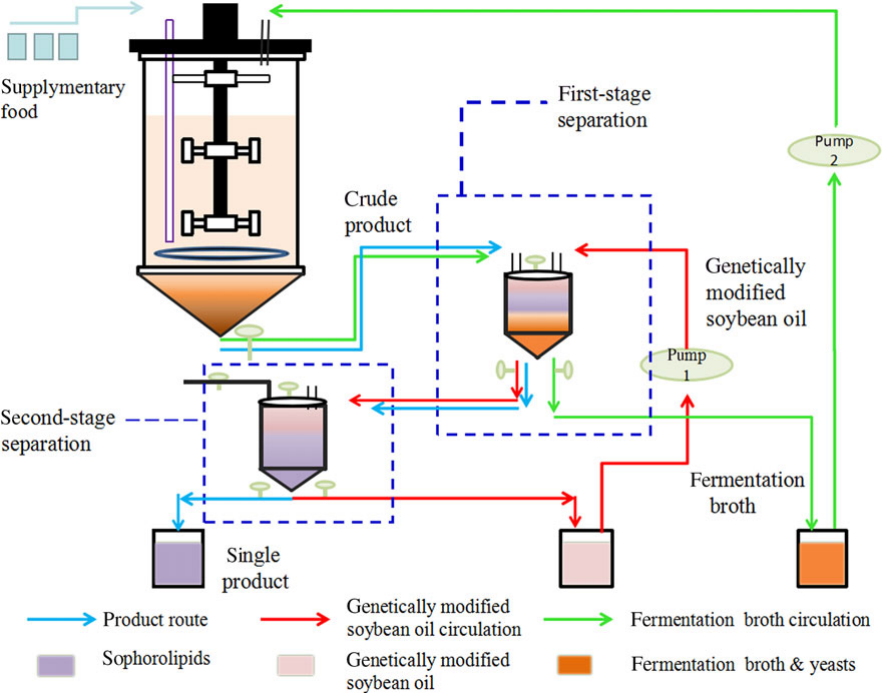
Coupled system of SL fermentation and separation. The blue arrow indicates the direction of product flow. The red arrow represents circulation of GMSO. The green arrow represents cycle utilization of fermentation broth. The dashed boxes represent first and second separation from top to bottom. The tanks from left to right were used for collection of SL, GMSO and fermentation broth.

Based on the aforementioned observations, a two‐stage separation system coupled to DVDSB was designed for improving the efficiency of SL production. The detailed procedure has been shown in Fig. 5. When the SLs sedimented to the bottom of DVDSB, they were discharged into the first‐stage separator. Under the layering effect of GMSO, the broth containing cells sedimented to the bottom of the first‐stage separator. Next, the hydrophobic phase containing SLs and GMSO was transferred into the second‐stage separator, where purified SLs were collected by allowing the mixture to stand for 15 min. Subsequently, the purified SLs were transferred to the single product tank. There were two circulations in the fermentation and separation system. Initially, the fermentation broth flowed out of the fermenter and into the first‐stage separator, and subsequently, it entered the fermentation broth‐collecting tank. Finally, the broth was transferred back into the fermenter for recycling. GMSO was pumped into the first‐stage separator from the oil‐storage tank, then into the second‐stage separator, and finally, back into the storage tank. The circulation routes combined the fermentation and separation processes together.

### Integrated process for semicontinuous fermentation and two‐stage separation

**Figure 6:**
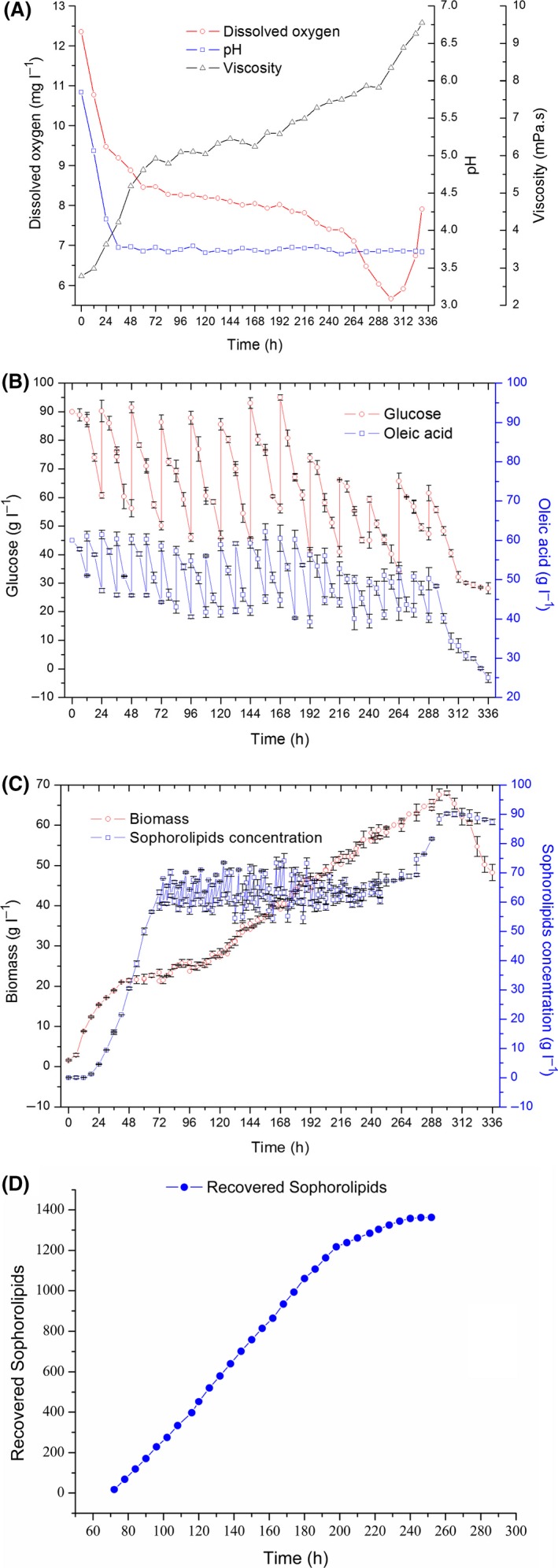
Fermentation curve during integrated semicontinuous fermentation and separation process over a 300‐h period. A. Time‐course of DO, viscosity and pH. B. Consumption of glucose and oleic acid substrates. C. Cell growth and SL production. D. Total amount of recovered SLs. The results shown are the mean values from triplicate experiments.

Semicontinuous fermentation was performed by integrating DVDSB and the two‐stage separation system. The experimental results are shown in Fig. 6.

During the first 48 h, both cell concentration and broth viscosity increased rapidly following increased DO consumption. Subsequently, the pH became acidic and was stabilized, while the viscosity and DO changed gradually, which provided a relatively stable external environment for SL production. The two‐stage separation system was activated at 70 h, when the SLs began to accumulate at the bottom of the fermenter. Crude SLs were discharged into the first‐stage separator, resulting in their separation from the fermentation broth, and transferred to the second‐stage separator for GMSO separation via static layering. The purified SLs were collected, while the fermentation broth, which essentially contained cells and the substrate, was recycled back into the fermenter. GMSO was also recycled and pumped into the first‐stage separator.

Compared with fermentation without the separation system, the maximum cell concentration increased from 61.76 to 67.96 g l^−1^ after integration of the separation system. High cell concentration was observed for about 200 h, which indicated that the concurrent product separation system considerably alleviated the problem of product inhibition and effectively recycled the broth. The total recovery of SLs was 1362 g as shown in Fig. 6d. Notably, SLs could not be discharged out of the fermenter after 252 h, as the increase in biomass resulted in high viscosity of the fermentation broth. The glucose consumption rate increased by 30%, from 1.31 to 1.71 g l^−1^ h^−1^. Particularly, at 300 h, SL concentration was observed to be 90.2 g l^−1^, while the productivity reached 1.59 g l^−1^ h^−1^. The above results suggested that coupling of semicontinuous fermentation and the two‐stage separation system was an effective strategy for improving SL production. Notably, the product purity was 74%, which was 23% higher than that observed for fermentation without the concurrent separation process. Our results showed that the separation system, resulting in the recycling of substrate and cells, was highly efficient in improving SL purification.

These results indicated that integration of the processes increased the efficiency of SL fermentation, by achieving concurrent separation and purification. In addition, the problem of product inhibition was considerably alleviated due to the timely discharge of SLs from the fermenter. In contrast to the semicontinuous fermentation with DVDSB, the production of SLs (477 g l^−1^) was similar to that in the actual condition of fermentation process. In the absence of a separation system, the production of SLs appeared to be high due to the loss of fermentation broth. However, the addition of a separation system recycled the fermentation broth, which ensured that the volume of broth in this fermenter was higher than that observed in the fermenter without a separation system. The process of SL production has been summarized in Table 1.

**Table 1:** Bioprocesses for SLs fermentation

Fermentation mode	Glucose (g l^−1^)	Oleic acid (g l^−1^)	Period (h)	Sophorolipids (g l^−1^)^a^	Biomass (g l^−1^)	Productivity (g l^−1^ h^−1^)	Product purity
Batch fermentation in conventional bioreactor	90	90	96	104.7	21.98	1.09	–
Fed‐batch fermentation in conventional bioreactor	120	120	96	120	24.02	1.25	–
Semicontinuous fermentation in new bioreactor with DVDSB	420	385	320	484	61.76	1.51	60%
Semicontinuous fermentation in DVDSB coupled with two‐stage separation system	510	380	300	477	67.96	1.59	74%

**a.** The production of sophorolipids was calculated by gross product/the final volume of fermentation broth.

Compared with the traditional batch fermentation systems, in our system with integrated fermentation and separation processes, SL production increased about four times (477 g l^−1^), while its productivity increased by 27% (1.59 g l^−1^ h^−1^). Further, after the integration of the two‐stage separation system, SL purity also increased from 60 to 74%. The two‐stage concurrent separation system coupled with the cell‐recycling DVDSB not only decreased the problem of product inhibition considerably, it also increased the efficiency of SL production. Thus, an integrated system was established by coupling semicontinuous fermentation with the two‐stage separation system for SL production by *C. albicans* O‐13‐1.

## Experimental procedures

### Strain, medium and culture conditions

*Candida albicans* O‐13‐1 was obtained from the Ocean University of China. Slant medium consisted of 20 g l^−1^ glucose, 10 g l^−1^ yeast extract, 20 g l^−1^ peptone and 20 g l^−1^ agar, with pH adjusted to 6. Seed medium consisted of 100 g l^−1^ glucose, 10 g l^−1^ yeast extract and 1 g l^−1^ peptone with pH adjusted to 6. The medium of batch fermentation consisted of 90 g l^−1^ glucose, 90 g l^−1^ oleic acid, 3.5 g l^−1^ yeast extract, 0.5 g l^−1^ peptone, 5 g l^−1^ sodium citrate, 4 g l^−1^ MgSO_4_·7H_2_O, 2 g l^−1^ (NH_4_)_2_SO_4_, 1 g l^−1^ KH_2_PO_4_, 0.1 g l^−1^ NaCl and 0.1 g l^−1^ CaCl_2_·2H_2_O, with pH adjusted to 6. Initial fermentation medium in the fed‐batch and semicontinuous bioreactors contained 90 g l^−1^ glucose and 60 g l^−1^ oleic acid, while the rest of the components were the same as those used for batch fermentation. Supplemented medium contained 5 g l^−1^ yeast extract and 2 g l^−1^ peptone.

### Batch and fed‐batch fermentation in traditional 5‐l bioreactor

A 500‐ml Erlenmeyer flask containing 100 ml seed medium was inoculated with 1 ml of 10^7^ per ml yeast suspension. The flask was incubated at 28–30°C, with shaking at 220 rpm for 48 h. The culture was then transferred into a 5‐l fermenter containing 3 l fermentation medium. The DO concentration in the broth was monitored using an online controller (BLBIO‐5GJ; Shanghai Bailun Bio‐Technology co., Ltd, Shanghai, CN, USA), and maintained at 30–50% by delivering sterile air into the bioreactor via a pipeline for oxygen supply. The conditions maintained with the fermenter were as follows: 10.5 l min g l^−1^ airflow rate, 450 rpm stirring speed, 30°C temperature and 1 kW per 220 V AC stirring power. For fed‐batch fermentation, the liquid level in the bioreactor was maintained by adding supplemented medium at 24‐h intervals. Oleic acid concentration was maintained at 30–60 g l^−1^ by adding oleic acid at 12‐h intervals, while glucose concentration was maintained at 30–90 g l^−1^ by adding 200 g l^−1^ glucose solution every 24 h. The pH was maintained at 5.8–6.2 during the first 20 h. Thereafter, the pH was maintained at 3.5–4, whereas the airflow rate, stirring speed and temperature were the same as those used for batch fermentation.

### Effect of added SLs on growth of *C. albicans* O‐13‐1

Sophorolipids gradient on slants: When the OD_600_ of *C. albicans* O‐13‐1 culture reached 0.5, the yeast cells were collected and washed thrice with sterile water. Serial dilutions of the yeast solution were prepared to obtain 1 ml working solutions containing from 10 to 10^−5^ cells ml^−1^. Each dilution was inoculated (1 μl) was inoculated onto slants containing SL with 0–95 g l^−1^ concentration gradient. Then, the petri dishes were incubated at 28–30°C for 48 h.

Inhibition test under fermentation conditions: Our laboratory produced a *UGTA1* gene knockout *C. albicans* O‐13‐1 strain that lost SL‐producing function. The *UGTA1* gene was deleted by inserting a hygromycin resistance (*HygR*) gene cassette in its codon sequence. The *HygR* CDS was cloned from pFA6*a*‐link‐yECitrine‐Hygro plasmid and placed between the promoter *Peno* and terminator T*pgk1*. The *HygR* gene expression cassette was then flanked by two homologous arms from *UGTA*1 with fusion polymerase chain reaction using the primers listed in Table 2. The deletion fragments were transformed as previously reported by Saerens *et al*. (2011a,b). The yeast cells (10^7^ per ml) were inoculated into a 100‐ml Erlenmeyer flask containing 30 ml seed medium and incubated at 28–30°C, with shaking at 220 rpm for 2 days until the medium turned milky. The seeds were inoculated into Erlenmeyer flasks containing 30 ml liquid slant medium with different concentration of SLs from 35–95 g l^−1^. SLs were separated from the fermentation broth using ethyl acetate and concentrated to remove the solvent by vacuum evaporation at 50°C. The OD_600_ of yeast cells was measured at 5 h intervals. The residual glucose and SL concentrations were measured every 12 h.

**Table 2:** Primers used for UGTA1 gene deletion

Prime name	Sequence (5′–3′)
UGTA1A‐F	CAGATATGCATCAGGGGCACAG
Peno–UGTA1A‐R	TATTCTGAGTGTTGTGAATTGAGTTCGGTGCTTG
UGTA1A –Peno‐F	ACCGAACTCAATTCACAACACTCAGAATACGAGTTTG
Hyg‐ Peno‐R	GCTTTTTACCCATTTCTAATAGATGTTTGTCTG
Peno–hyg‐F	CATCTATTAGAAATGGGTAAAAAGCCTGAACTCAC
Tpgk1‐Hyg‐R	CTGCACATACCAGTTTATTCCTTTGCCCTCGGACGAG
Hyg‐Tpgk1‐F	GAGGGCAAAGGAATAAACTGGTATGTGCAGTCATCTCT
UGTA1B‐ Tpgk1‐R	CTAAGAACTCACCGCATAACGTTCATGGTTACTATG
Tpgk1‐ UGTA1B‐ F	CCATGAACGTTATGCGGTGAGTTCTTAGAATCGTAC
UGTA1B‐R	TTGACCAGGAAATGGTCTCTTG

### Two‐stage separation system

A two‐stage separation system was added to DVDSB in semicontinuous SLs fermentation. The system mainly consisted of first‐ and second‐stage separators, a pumping device for GMSO, and a reflux device for the broth. (Fig. 5) The biological separator was funnel‐shaped for sampling. (Fig. S4) Other parts of the separation system included pressure balanced valves, reflux route pumps, pipelines connecting different parts of the separating equipment and valves to control switches. In the first‐stage separation process, GMSO was selected as the phase separation agent to promote SL separation from the broth. SLs were further separated with GMSO by settling during the second‐stage separation process. The system also allowed for recycling of substrates and yeast cells.

### Semicontinuous fermentation process in DVDSB coupled with the two‐stage separation system

The specific configuration and design principle of the novel bioreactor DVDSB are shown in Fig. S5 and Appendix S1. As for semicontinuous fermentation in DVDSB coupled with the novel separated system, the seeding medium, fermentation medium and feeding methods were the same as those used for fed‐batch fermentation. The pH value was maintained within 5.8–6.2 during the first 20 h, and subsequently maintained at 3.5–4. The airflow rate was maintained at 8 l min^−1^ and stirring speed between 350 and 450 rpm. Supplemented medium contained 200 g l^−1^ glucose, 5 g l^−1^ yeast extract and 2 g l^−1^ CO(NH_2_)_2_. The effective volume of the novel bioreactor was 5.7 l, while the initial liquid volume in the fermenter was 3.5 l (without oleic acid).

The separation system (Fig. S6) was concurrent during semicontinuous fermentation using DVDSB. SLs began to accumulate at 70 h. The lower ventilator was closed every 4–6 h; thus, allowing crude SLs to deposit at the bottom of the tank. Subsequently, the valve at bottom of the DVDSB was opened to let the crude SL enter the first‐stage separator. Effected by GMSO, the mixture divided into two phases within 1 min. The fermentation broth containing cells remained within the lower phase and flowed into the broth‐collecting tank. The SLs and GMSO floated in the upper phase, which was transferred to the second‐stage separator by opening the relevant valves. After resting for 15 min in the second‐stage separator, SLs were separated using GMSO and collected in the product tank. The broth containing cells was pumped to the DVDSB, while GMSO was pumped into the first‐stage separator, to be recycled. The design principle and detailed operating instructions of the two‐stage separation system are shown in Appendix S1.

### Analytical methods

The biomass, residual glucose, oil, viscosity of fermentation broth and SL concentration were measured using 10 ml of fermentation broth. The biomass was measured as previously reported by Pan *et al*. (2012). The cell culture growth rate during product inhibition was determined by measuring the OD_600_ using a spectrophotometer (Cary 50 UV–vis; Shanghai Aplish co., Ltd, Shanghai, CN, USA). SL content was calculated by deducting residual glucose content from total sugar content. Total sugar in the crude product or fermentation broth was quantified by the anthrone method (Buschmann *et al*., 1996; Ma *et al*., 2011). The anthrone method (total sugar quantification): 0.5 ml diluted crude product separated by the separation system (or 0.5 ml broth) was washed thrice using equal volumes of hexane to remove oil and fat. SL was dissolved by adding 1 ml acetonitrile to 0.5 ml diluted crude product (or broth) and the solution was centrifuged at 10 000 rpm for 10 min. Residual glucose content was determined using bio‐sensor SBA‐90 (Biology Institute of Shandong Academy of Science, Shandong, CN, USA), calibrated by 100 mg dl^−1^ glucose solution. The crude product sample (or broth) was diluted to an appropriate range (10–150 mg dl^−1^). The injection volume was 25 μl, and the residual glucose content was the product of the result obtained by the qualitative method and the dilution ratio.

The fermentation system screen readout provided the DO and pH values (Yang *et al*., 2012). Residual oleic acid was measured qualitatively. In brief, the fermentation broth sample was centrifugalized at 5000 rpm and oleic acid in upper phase was extracted using organic solvents in a dryer at 60°C. Viscosity was estimated using the NCY‐6 full‐automatic viscometer (Shanghai S.R.D. scientific instrument CO., LTD, Shanghai, CN, USA), by injecting 25 ml fermentation broth into the capillary viscometer of the full‐automatic equipment, at constant temperature. Each assay was repeated three times, and the average values are presented.

## Conflict of interest

None declared.

## Supporting information


**Fig. S1.** Sophorolipid (SL) production using different concentrations of carbon source.
**Fig. S2.** Inhibitory effect of added SLs on growth of *Candida albicans* O‐13‐1 in petri dishes.
**Fig. S3.** Miscibility and stratification properties of GMSO, SL and fermentation broth.
**Fig. S4.** Schematic of the newly designed separator.
**Fig. S5.** Schematic of the new bioreactor with dual ventilation pipes and dual sieve‐plates (DVDSB).
**Fig. S6.** Schematic of the two‐stage separation system.
**Table S1.** Optimization of fermentation conditions in the 5‐l Batch fermentation.
**Table S2.** Optimization of fermentation medium in the 5‐LBatch fermentation.
**Appendix S1.** Experimental procedures.
