# Supplementary material for: Semicontinuous sophorolipid fermentation using a novel bioreactor with dual ventilation pipes and dual sieve‐plates coupled with a novel separation system
Source: Microb Biotechnol. 2017 Dec 13;11(3):455–64. doi: 10.1111/1751-7915.13028 (PMC5902327; doi:10.1111/1751-7915.13028)
Supplement: Supplementary file 1 — Fig. S1. Sophorolipid (SL) production using different concentrations of carbon source. Fig. S2. Inhibitory effect of added SLs on growth of Candida albicans O‐13‐1 in petri dishes. Fig. S3. Miscibility and stratification properties of GMSO, SL and fermentation broth. Fig. S4. Schematic of the newly designed separator. Fig. S5. Schematic of the new bioreactor with dual ventilation pipes and dual sieve‐plates (DVDSB). Fig. S6. Schematic of the two‐stage separation system. Table S1. Optimization of fermentation conditions in the 5‐l Batch fermentation. Table S2. Optimization of fermentation medium in the 5‐LBatch fermentation. Appendix S1. Experimental procedures. [file MBT2-11-455-s001.doc]

**Supplementary Information**

**Supplementary Tables**

**Table S1 Optimization of fermentation conditions in the 5-l Batch fermentation.^a^**

Optimize results of fermentation conditions (pH, temperature, inoculation quantity and stirring speed) in the 5-L batch fermentation via orthogonal optimization tests

| Factor | pH | Temperature  (℃) | Inoculation quantity (%) | Stirring speed  (rpm) | DCW  (g/l) | Production  (g/l) | Yield  (g/g) |
| --- | --- | --- | --- | --- | --- | --- | --- |
| a | 1(5.5) | 1(22) | 1(3) | 1(300) | 20.03±0.99 | 90.51±1.22 | 0.50±0.03 |
| b | 1 | 2(26) | 2(5) | 2(450) | 19.82±1.36 | 71.15±2.25 | 0.40±0.04 |
| c | 1 | 3(30) | 3(7) | 3(600) | 17.97±1.75 | 45.57±0.78 | 0.25±0.01 |
| d | 2(6.0) | 1 | 2 | 3 | 18.24±2.04 | 46.85±0.91 | 0.26±0.02 |
| e | 2 | 2 | 3 | 1 | 20.16±1.77 | 98.80±0.88 | 0.55±0.02 |
| f | 2 | 3 | 1 | 2 | 20.01±2.28 | 76.55±1.82 | 0.43±0.04 |
| g | 3(6.5) | 1 | 3 | 2 | 18.89±2.09 | 73.98±1.79 | 0.41±0.01 |
| h | 3 | 2 | 1 | 3 | 16.17±1.16 | 38.86±2.05 | 0.22±0.03 |
| j | 3 | 3 | 2 | 1 | 21.12±2.44 | 96.75±1.63 | 0.54±0.02 |

^a^ Values are the averages of triplicate experiments ± SD.

**Table S2** **Optimization of fermentation medium in the 5-LBatch fermentation.^a^**

Optimize results of fermentation medium (glucose, yeast powder, peptone, oleic acid and different concentrations of the substrates) in the 5-L batch fermentation via orthogonal optimization tests.

| Factor | Glucose  (g/l) | Peptone  (g/l) | Oleic acid (g/l) | Yeast powder  (g/l) | DCW  (g/l) | Production  (g/l) | Yield(g/g) |
| --- | --- | --- | --- | --- | --- | --- | --- |
| a | 1(80) | 1(0.5) | 1(80) | 1(2.5) | 19.22±0.78 | 78.5±0.56 | 0.49±0.01 |
| b | 1 | 2(0.7) | 2(90) | 2(3) | 19.28±1.33 | 74.8±0.92 | 0.44±0.01 |
| c | 1 | 3(0.9) | 3(100) | 3(3.5) | 18.54±2.16 | 60.5±1.31 | 0.34±0.03 |
| d | 2(90) | 1 | 2 | 3 | 20.75±1.47 | 98.2±1.25 | 0.55±0.02 |
| e | 2 | 2 | 3 | 1 | 20.81±1.91 | 93.2±2.01 | 0.49±0.03 |
| f | 2 | 3 | 1 | 2 | 20.43±2.22 | 80.7±2.40 | 0.47±0.04 |
| g | 3(100) | 1 | 3 | 2 | 21.14±2.41 | 99.5±1.76 | 0.50±0.02 |
| h | 3 | 2 | 1 | 3 | 21.56±1.72 | 93.5±0.99 | 0.52±0.01 |
| j | 3 | 3 | 2 | 1 | 21.98±0.42 | 102.5±0.85 | 0.54±0.02 |

^a^ Values are the averages of triplicate experiments ± SD.

**Supplementary Figures**

**Fig. S1 Sophorolipid (SL) production using different concentrations of carbon source.**

Glucose:Oleic acid (g:g) = 90:60 indicates 90 g/L glucose and 60 g/L oleic acid used in the fermentation broth. Graph represents average ± SD from triplicate experiments.

**
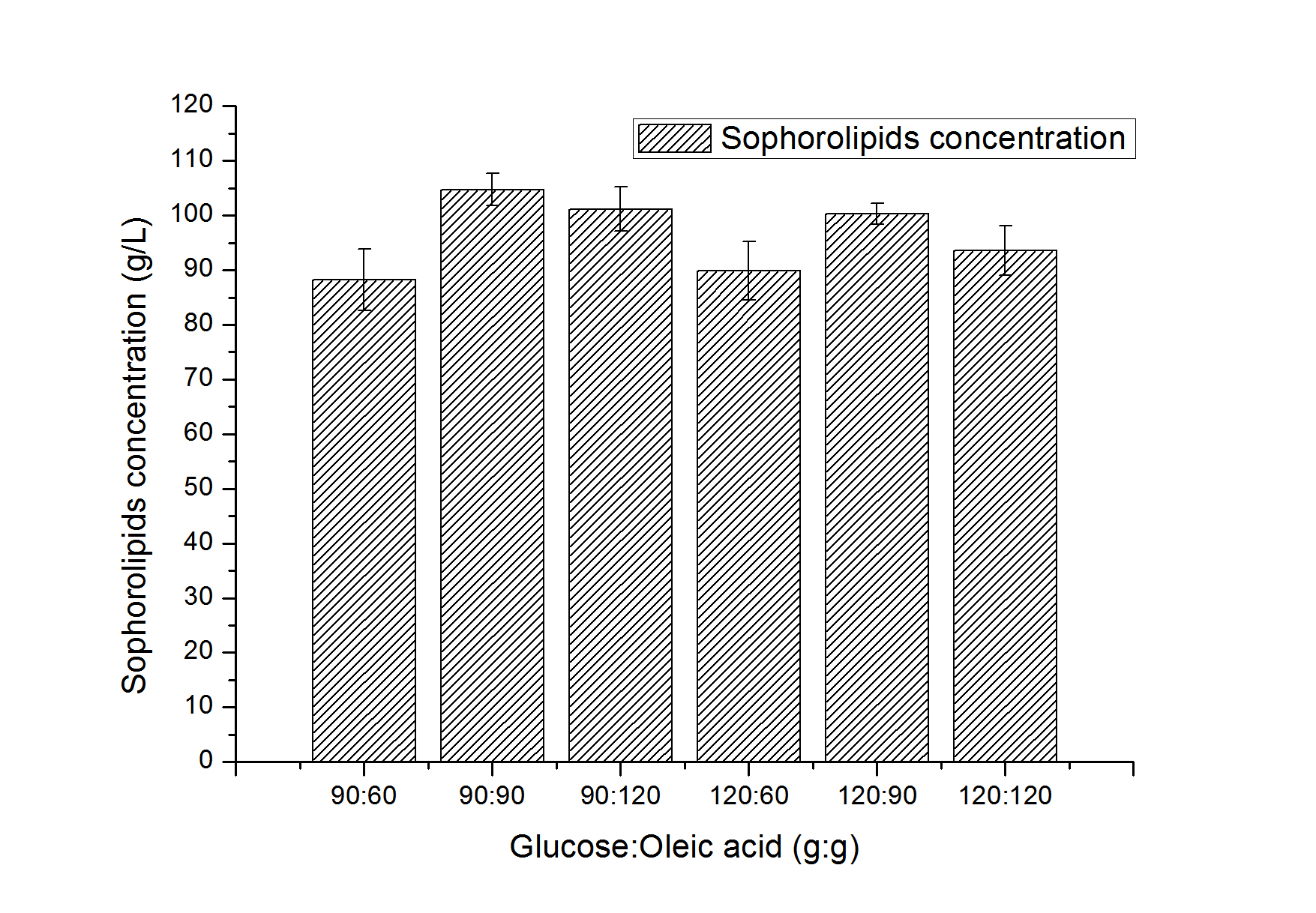
**

**Fig. S2**

**Inhibitory effect of added SLs on growth of *Candida albicans* O-13-1 in petri dishes.**

An SL gradient from 0 to 95 g/L was incorporated in the slant medium in petri dishes. Yeast culture solution with OD600 of 0.5 was serially diluted from 10 to 10^-5^ and each dilution was added into a separate petri dish.


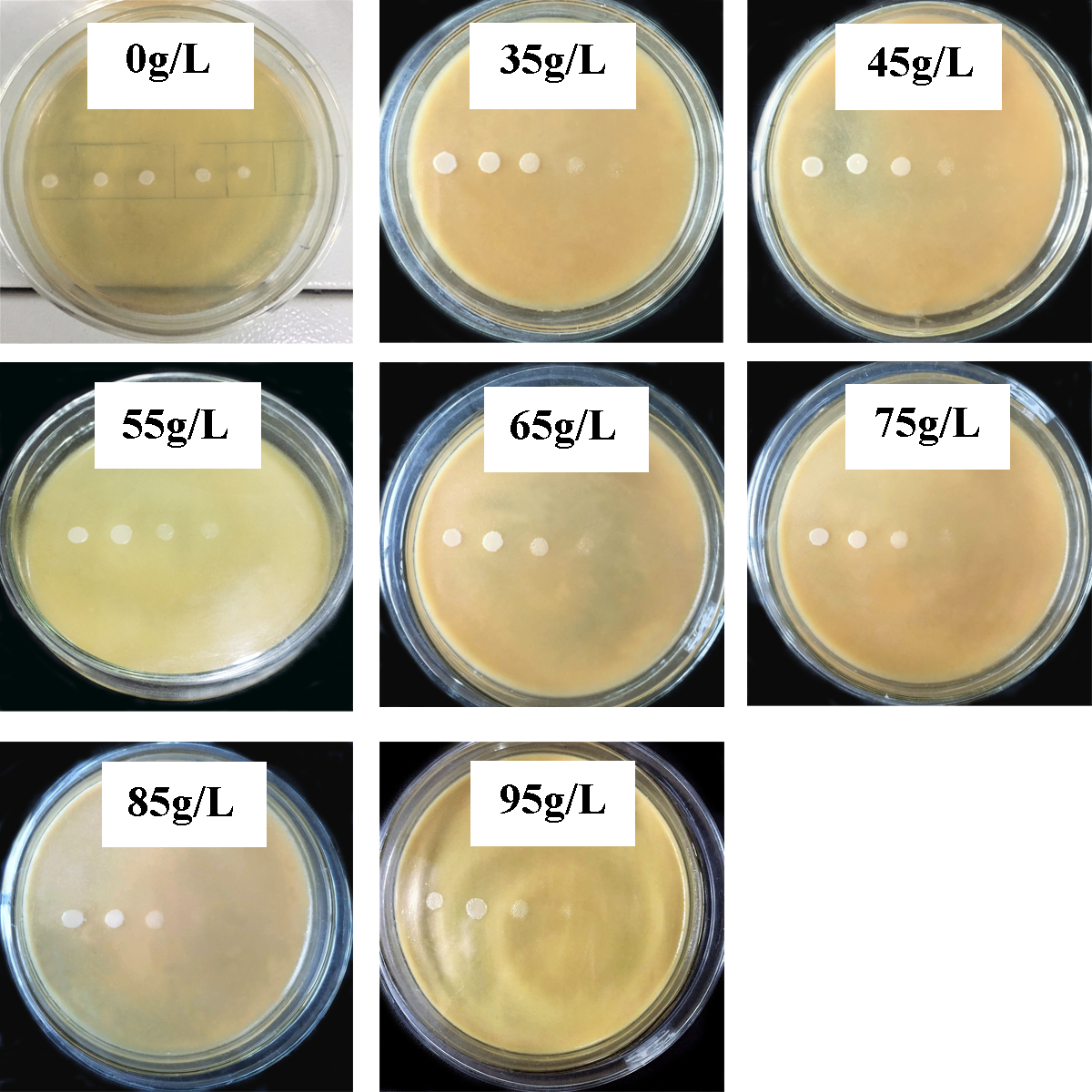


**Fig. S3A**

**Miscibility and stratification properties of GMSO, SL and fermentation broth.**

Different ratios of GMSO to SL (5:1, 2:1, 1:1, 1:2, and 5:1) were selected to determine the ability of GMSO to promote layering of fermentation broth and SL. GMSO and SL in different proportions were added into reagent bottles containing 20 mL broth. In each reagent bottle, total quantity of GMSO and SL was 36 g.

**
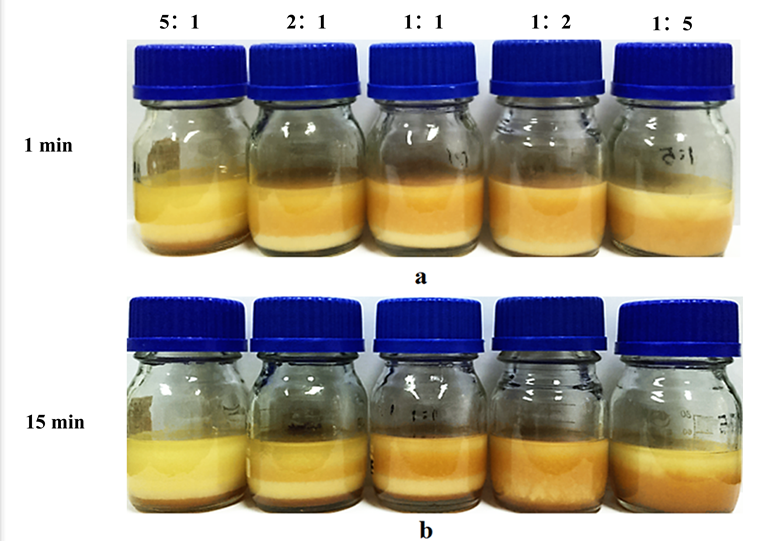
**

**a** 1 min after the mixing of GMSO, SLs and fermentation broth. From left to right, the ratios of GMSO

and SLs are 5:1, 2:1, 1:1, 1:2 and 1:5. **b** 15-30 min after the mixing of GMSO, SLs and fermentation broth. From left to right, the ratios of GMSO and SLs are 5:1, 2:1, 1:1, 1:2 and 1:5.

**Fig. S3B**

**Fig. S3B**

**Miscibility and stratification properties of conventional soy bean oil (SBO), SL and fermentation broth.**

Different ratios of SBO to SL (5:1, 2:1, 1:1, 1:2, and 5:1) were selected to determine the ability of SBO to promote layering of fermentation broth and SL. GMSO and SL in different proportions were added into reagent bottles containing 20 mL broth. In each reagent bottle, total quantity of GMSO and SL was 36 g.

**
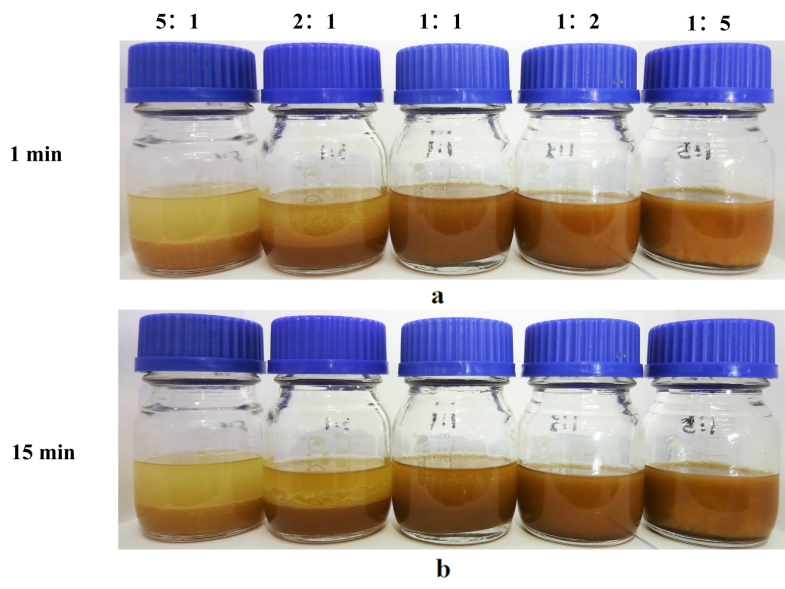
**

**a** 1 min after the mixing of SBO, SLs and fermentation broth. From left to right, the ratios of SBO and SLs are 5:1, 2:1, 1:1, 1:2 and 1:5. **b** 15-30 min after the mixing of SBO, SLs and fermentation broth. From left to right, the ratios of SBO and SLs are 5:1, 2:1, 1:1, 1:2 and 1:5.

**Fig. S4 Schematic of the newly designed separator**

**
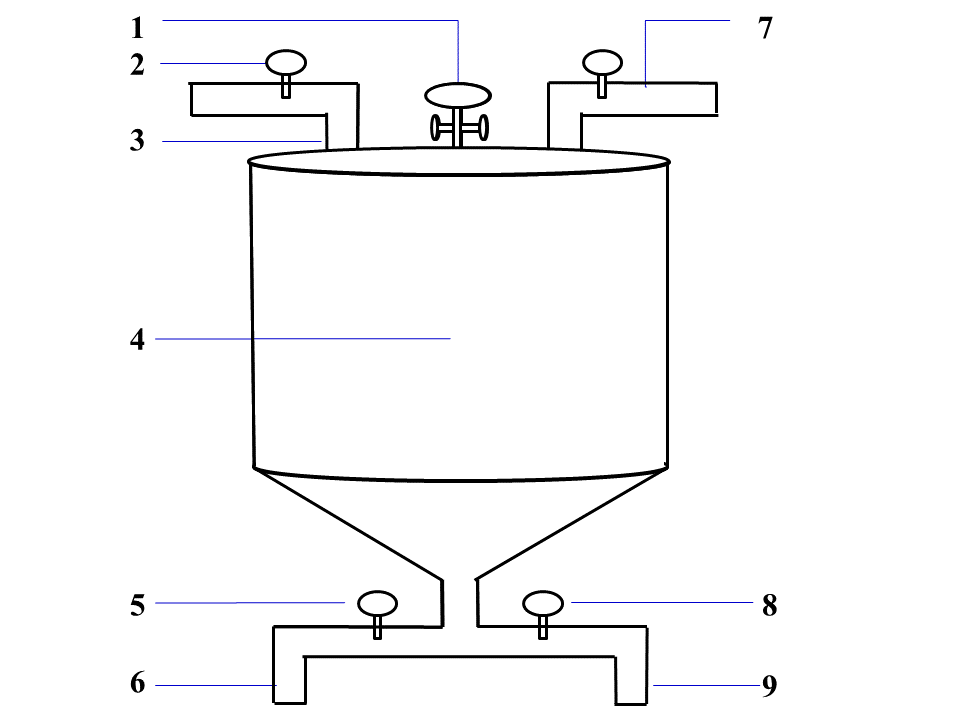
**

**(1****)** pressure balanced two-way valve; **(4)** separation tank; **(3)&(7)** the first and second feeding pipelines; **(6)&(9),** the first and second split-phase pipelines; (**2), (5)&(8)** valves.

**Fig. S5 Schematic of** **the new bioreactor with dual ventilation pipes and dual sieve-plates (DVDSB).**

**
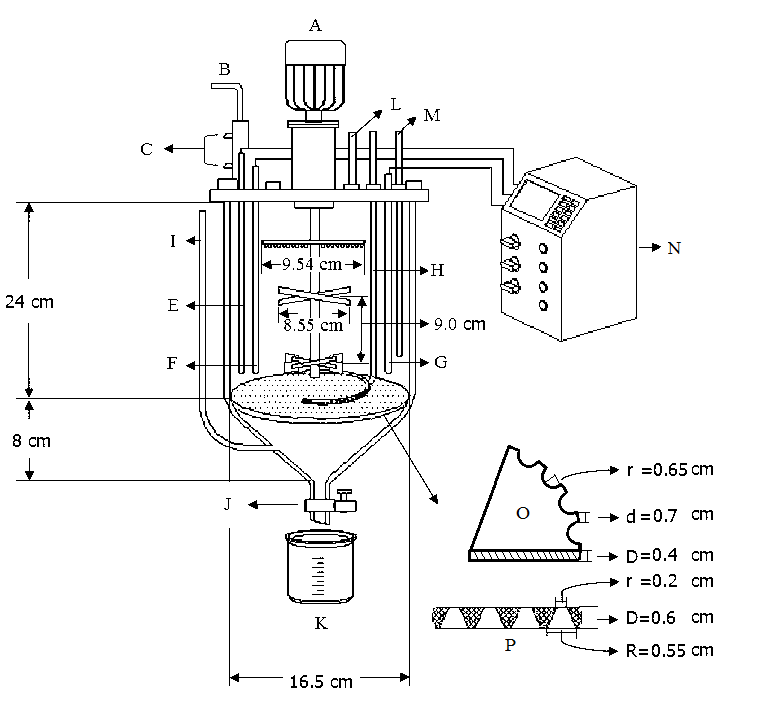
**

**A**, motor and stirrer; **B**, exhaust port; **C**, water circulation line; **E**, pH electrode; **F**, temperature electrode; **G**, dissolved oxygen electrode; **H**, assistant oxygen supply pipeline; **I**, main oxygen supply pipeline; **J**, discharge pipe; **K**, product storage tank; **L**, feed pipe; **M**, sampling pipe; **N**, control unit; **O**, partial, enlarged view of sieve plate A; and **P**, partial, enlarged view of sieve plate B.

**Fig. S6 Schematic of the two-stage separation system**

**
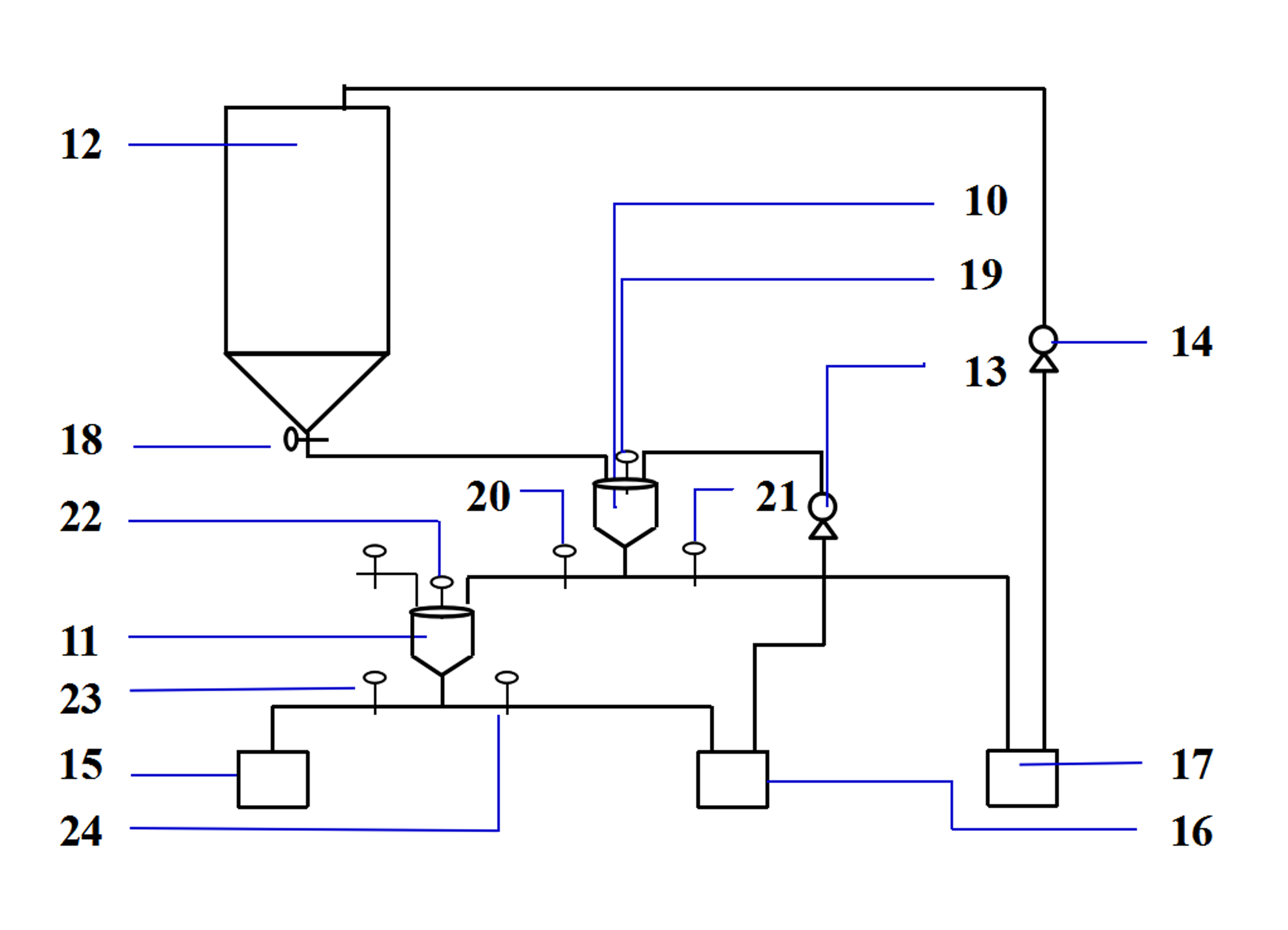
**

(**10**) the first-stage separator; (**11**) the second-stage separator; (**12**) fermenter; (**13**) the first pump; (**14**) the second pump; (**15**) purified product tank; (**16**) GMSO collecting tank; (**17**) fermentation broth collecting tank; (**19**) **&** (**22**) pressure balanced valves; (**18**)**,** (**20**)**,** (**21**)**,** (**24**) **&** (**25**) valves.

**Supplemental Experimental Procedures**

**Design of the bioreactor with dual ventilation pipes and dual sieve-plates (DVDSB).**

The configuration of DVDSB is shown in Fig. S5. The sieve plate divides the tank into a cylindrical area and a cone-shaped area. Fermentation occurs in the cylindrical area, and the SLs sediment in the cone-shaped area. The cylinder-to-cone volume ratio is 9:1. The height-to-diameter ratio of the cylindrical area is 3:2. The sieve plate contains two layers: an upper circular plate with semicircular grooves at the edge, and a lower polyporous plate. The upper layer is made of flexible soft silicone. The lower layer, which is required for support, is fabricated from hard glass. The two layers of the sieve plate are easily separated and combined by controlling the airflow. Other components include the exhaust port, water circulation line, inoculum entrance, sampling line, [feed pipe](app:ds:feed pipe), motor, stirrer, and the pH, temperature, and dissolved oxygen electrodes.

In addition, there are two ventilation pipelines in this novel bioreactor. The main oxygen supply pipeline is in the cone area, while the auxiliary oxygen pipeline is in the cylindrical area. The main and auxiliary oxygen supply pipelines provide oxygen for the entire bioreactor and the cylindrical area, respectively.

**Design of the integrated fermentation and separation system**

As shown in Fig. S6, the bottom of the fermenter (**12**) is connected with the first valve (**18**) and upper wall of the first-stage separator (**10**) through pipelines. This is required for transfer of the crude product from the fermenter to the first-stage separator. The GMSO tank (**16**) is connected with the first pump (**13**) and the first-stage separator via pipelines, which is required for the transfer of GMSO to the first-stage separator. Pipelines at the bottom of the first-stage separator are connected in two ways. First, they are connected with the valve (**20**) and top of second-stage separator (**11**), which is required for the transfer of the GMSO and SL phase into the second-stage separator. Second, they are connected with the first pump (**21**), fermentation broth tank (**17**), the second pump (**14**), and the top wall of the fermenter (**12**), which is required for the collection of fermentation broth at a lower level. The second-stage separator is connected with the single product (**15**) and GMSO (**16**) tanks via two pipelines (valves **23** and **24**), respectively. The purified product and GMSO (after use) were collected. The top walls of the first- and second-stage separators are connected to the pressure balance valves **19** and **22,** respectively, via pipes. The pressure balance valves are connected on the top caps of separators. The fermentation and separation equipment is maintained under aseptic conditions. In the above description, the numbers in bold represent the different parts of the integrated system as shown in Figure S6.

**Operating instructions for the integrated fermentation and separation system**

As shown in Fig. S6, the method for operating the fermentation–separation system is as follows: Open the first valve (**18**) and let the crude product enter the first-stage separator (**10**) from the fermenter (**12**). Pump the stored GMSO into the first-stage separator by launching the first pump (**13**). The mixture gets divided into two phases within 1 min. The upper phase contains a mixture of GMSO and SLs, while the lower phase contains the fermentation broth. Adjust the first pressure balancing valve (**19**) and allow the fermentation broth to enter the fermentation broth collecting tank by closing the second valve (**20**) and opening the third valve (**21**). The fermentation broth enters the fermenter (**12**) via the second pump (**14**). The mixed phase of GMSO and SLs flows into the second-stage separator by closing the third valve (**21**) and opening the second valve (**20**). After allowing to stand for 15 min, the SLs and GMSO distribute between different two phases. The second pressure balancing valve **22** is adjusted to balance the air pressure inside the second-stage separator. SLs enter the single product tank (**15**), when the fourth valve (**23**) is closed and the fifth valve **24** is opened. GMSO enters the tank (**16**) by an opposite mechanism. GMSO and the fermentation medium can be recycled in the fermentation and separation system.
